# Assessing the use of antibiotics and the burden of varicella in Belgium using a retrospective GP database analysis

**DOI:** 10.1186/s12879-021-06848-4

**Published:** 2021-11-11

**Authors:** Jessica Vandenhaute, Elyonore Tsakeu, Pierre Chevalier, Manjiri Pawaskar, Goran Benčina, Jan Vertriest

**Affiliations:** 1 MSD, Clos du Lynx 5, Sint-Lambrechts-Woluwe, 1200 Brussels, Belgium; 2 Real World Evidence, IQVIA, Zaventem, Belgium; 3 Center for Observational and Real-World Evidence, Merck & Co., Inc, Kenilworth, NJ USA; 4 MSD, Madrid, Spain

**Keywords:** Antibiotics, Varicella, Complications, Primary care, Burden of varicella

## Abstract

**Background:**

Varicella is a highly contagious infection that typically occurs in childhood. While most cases have a generally benign outcome, infection results in a considerable healthcare burden and serious complications may occur.

**Objectives:**

The objective of this study was to characterize the burden of varicella in a real-world primary care setting in Belgium, including the rate of varicella-related complications, medication management and general practitioner (GP) visits.

**Methods:**

The study was a retrospective observational study using data from a longitudinal patient database in a primary care setting in Belgium. Patients with a GP visit and a varicella diagnosis between January 2016 and June 2019 were eligible and data one month prior and three months after the diagnosis were included. Outcomes included varicella-related complications, antibiotic use, antiviral use, and GP follow-up visits. Antibiotic use could be specified by class of antibiotic and linked to a diagnosis. Complications were identified based on concomitant diagnosis with varicella during the study period.

**Results:**

3,847 patients with diagnosis of varicella were included, with a mean age of 8.4 years and a comparable distribution of gender. 12.6% of patients with varicella had a concomitant diagnosis of a varicella-related complication. During the follow-up period, 27.3% of patients with varicella were prescribed antibiotics, either systemic (19.8%) and/or topical (10.3%). The highest rate of antibiotic prescriptions was observed in patients with complications (63.5%) and in patients younger than 1 year (41.8%). Nevertheless, 5.3% of the patients were prescribed antibiotics without a concomitant diagnosis of another infection. The most commonly prescribed systemic antibiotics were amoxicillin alone or combined with beta-lactamase inhibitor, and thiamphenicol. Fusidic acid and tobramycin were the most prescribed topical antibiotics. Antivirals were prescribed for 2.7% of the study population. 4.7% of the patients needed a follow-up visit with their GP.

**Conclusions:**

This study reports a substantial burden of varicella in a primary care setting in Belgium, with high rates of complications and antibiotic use.

**Supplementary Information:**

The online version contains supplementary material available at 10.1186/s12879-021-06848-4.

## Introduction

The Varicella zoster virus (VZV) is a highly contagious virus [1]. A primary infection with the virus causes varicella (chickenpox). This usually occurs during childhood. After the primary infection, the virus remains latent in the sensory nerve ganglia. The virus can be reactivated due to the waning cellular immunity to VZV (due to aging or some immunosuppressing states) causing herpes zoster (HZ) [2].

Live-attenuated vaccines against varicella have been available since 1970s [3]. However, its inclusion in routine vaccination programs varies across countries. Universal varicella vaccination (UVV) is currently recommended in 12 European countries, while in only seven (Finland, Germany, Greece, Hungary, Italy, Latvia and Spain) it is implemented and publicly funded. In other countries, such as United Kingdom and France, varicella vaccination is only recommended for high risk groups [4]. In most countries without UVV, the decision not to introduce a UVV program has been primarily based on varicella being considered as mild disease from a clinical and economic perspective, therefore associated with a low disease burden [4]. Another argument against UVV is the potential shift in the incidence of varicella disease in older ages, which might conversely result in increased morbidity and mortality in adults, despite a reduction in the number of paediatric varicella cases [4]. However, no indication of an age shift has been observed after implementation of UVV in several countries [4, 5]. In addition, an increase in the incidence of HZ due to exogenous boosting is another argument not to implement UVV. The theory of exogenous boosting hypothesizes that re-exposure to wild circulating varicella virus can boost the cell-mediated immunity, and, as a result, could prevent the development of HZ later in life. This would imply that UVV could lead to an increase of the HZ incidence. This hypothesis was generated in the mid-1960s [6]. However, recent data are inconclusive in terms of impact of UVV on exogenous boosting and HZ incidence [5, 7–9].

In most cases, varicella is a self-limiting, mild disease. It usually presents as a vesicular eruption associated with general symptoms like fever and malaise [10]. Nevertheless, varicella can result in severe complications leading to hospitalisation and comorbidities. These can range from dehydration and skin infections to pneumonia and encephalitis [11]. It is reported that 2–6% of patients with varicella infection attending general practice develop complications [12, 13]. In Belgium, the estimated incidence of varicella is around 113,000 cases per year, which results in 28.8–35.7 general practitioner (GP) visits per 10,000 patients [12, 14, 15]. Moreover, the incidence of varicella-related paediatric hospitalisation is 29.5/100,000 person-years, including 19/100,000 person-years of complicated hospitalised cases. The mortality rate of varicella in Belgium among patients younger than fifteen is 0.05 per 100,000 [12, 13].

Despite the fact that in most cases, individual disease burden is mild, the socio-economic burden of varicella in Europe is substantial. Wysocki et al. 2018 estimated annual direct costs of varicella of almost €10 million with indirect costs of over €32 million in Poland alone in 2015 among children younger than 16 years. Average outpatient costs per varicella case were estimated at €245 and inpatient costs at €1198 [16]. In Germany, the economic burden of varicella was estimated to be €188 million in 2003. This was mainly driven by indirect costs due to loss of workdays by the parents [17]. The average number of workdays lost for adult cases of varicella, corrected for the employed population varied from 4.7 to 26.1 days while it ranged between 0.6 and 3.7 days for children (corresponding to the workdays lost to take care of children) [17].

Next to the substantial economic burden related to varicella infections, another global concern has risen, namely the extensive use of antibiotics and the rise of antimicrobial resistance. To this end, many countries, including Belgium, have undertaken multiple awareness campaigns targeting (in)appropriate use of antibiotics [18]. Nevertheless, the prescription of antibiotics for infections with a probable viral origin, such as acute respiratory tract infections, remains common [19]. In line with these observations, a recent study reported that antibiotics are regularly prescribed for the management of varicella-related complications. Antibiotics prescriptions also occurred without any mention of complications or a microbiological confirmation [20]. Vaccination can be one of the measures to help reduce the use of antibiotics via the prevention and control of infections [18]. To understand the clinical burden of varicella and reduce antibiotic use in children, this study aimed to increase insights in antibiotic use in varicella.

In this context, the primary aim of this study was to analyse the rate and patterns of antibiotics use for management of varicella and varicella-related complications in real-world primary care settings in the absence of UVV in Belgium. Burden of varicella in terms of complications, antiviral use, and number of GPs visits was also assessed as a secondary aim of the study.

## Methods and materials

The study was a retrospective observational study using data extracted from the IQVIA Longitudinal Patient Database (LPD), a database that has been widely used in previous drug utilization and epidemiological studies and represents a robust source of information on primary care in Belgium [21–23]. It is a dataset of pseudonymized electronic medical records (EMR) for over 3 million current and former patients. The panel includes a permanent and stable sample of 300 GPs and is nationally representative in terms of geographical coverage and patient demographics (gender and age). Given that a total of 13,661 GPs was active in Belgium in 2017, the database provides a coverage of approximately 2.5% of the Belgian GP community, accounting for about 460,557 patients associated with these GPs over the course of the year. This patient-level database captures patient demographics, diagnoses (using a specific diagnostic coding system that can be bridged with ICD-10-CM codes), medical history, prescriptions (associated with a hard-coded diagnosis) and other additional data such as height, weight, blood pressure and laboratory tests. All patients and GPs in the database are pseudonymized and can be followed longitudinally based on a unique identifier (ID). Strict attention to confidentiality is present at every stage of data collection, storage and analysis in accordance with GDPR and Belgian Ethics Committees recommendations.

**Fig. 1:**
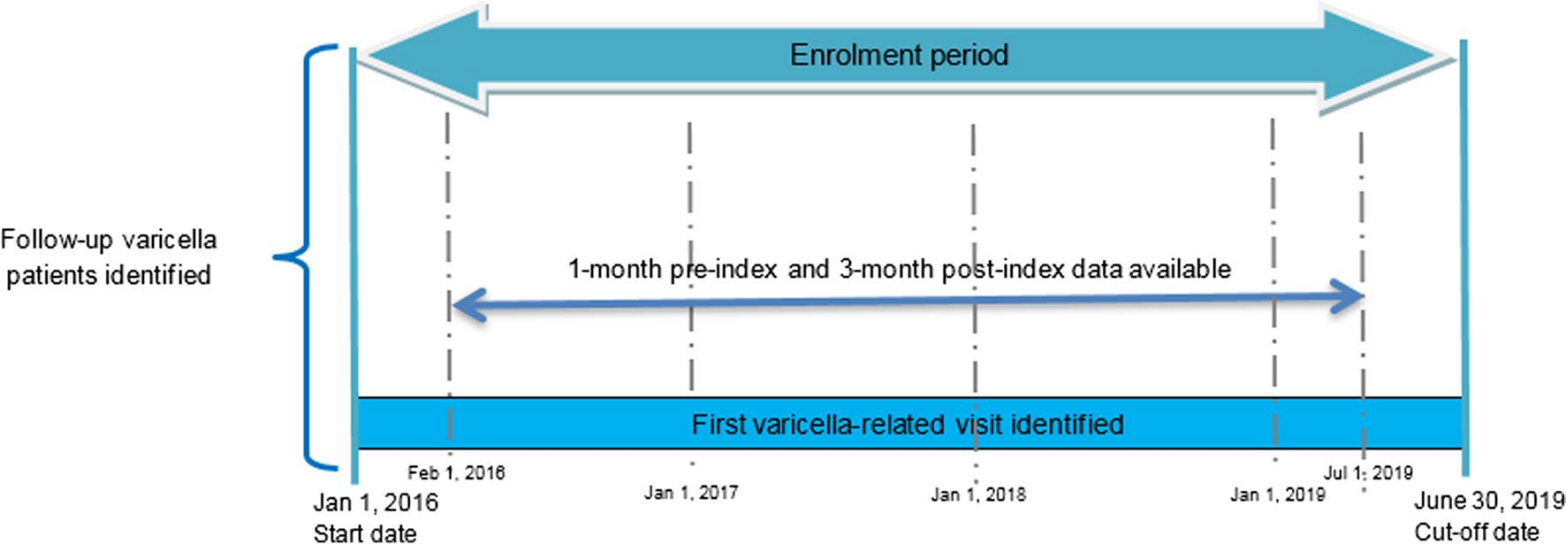
Diagram of patients’ selection in the primary care setting

The study period was defined as between January 1st 2016 and June 30th 2019. Cases were defined as: all patients with a GP visit and a diagnosis of varicella (ICD-10 code B01, as reported by GP) documented at any time over the study period (initial selection). The first varicella-related GP visit was identified as *index-visit* and the corresponding date was defined as the *index-date*. To guarantee continuity of patient data, additional eligibility criteria were included: (a) having at least one month of data available in the study period prior to the index-visit; (b) having at least three months of data available in the study period after the index-visit (Fig. 1). Patients were excluded if they had previously been vaccinated for varicella, i.e. if they received a dose of varicella live attenuated vaccine (identified by a prescription for a product in the ATC category J07BK01) at any time prior to the index-date.

For the selected patients, the ID was used to retrieve all the visits, both varicella and non-varicella-related, documented over the study period. All antibiotics received between 1-month pre-index and 3-months post-index were retrieved: systemic antibiotics were selected using ATC code J01 (antibiotics for systemic use) while topical antibiotics were selected using ATC codes D06A (antibiotics for topical use), R02AB (antibiotics for respiratory system) and S01AA (ophthalmological antibiotics). As it is mandatory for the physician to hard-code a diagnosis next to every prescription when completing the EMR, it was possible to differentiate between antibiotics prescribed for potential varicella-related complications or other diagnoses.

Varicella-related complications were captured during the index-visit or in any other visit recorded between 1-month pre-index and 3-months post-index to account for a potential delayed confirmation of varicella diagnoses associated with varicella-related complications. The complications were identified according to two strategies. The first strategy consisted in looking at the presence of additional digits to the main varicella code, which can be used to code complications, i.e. varicella meningitis as B01.0; varicella encephalitis, myelitis and encephalomyelitis as B01.1; varicella with pneumonia as B01.2; varicella with other complications as B01.8; and varicella without complications as B01.9. However, GPs tend to not systematically use these digits and to code complications separately. Therefore, in order to avoid a systematic underestimation of the rate of complication, a second approach was also taken, consisting of looking for concomitant diagnostic codes distinct from varicella but corresponding to common complications associated with varicella. A list of ICD-10-CM codes was compiled from the studies of Blumental et al. [12] and Wolfson et al. [20], resulting in the following diagnoses to be searched: varicella meningitis, varicella encephalitis, myelitis and encephalomyelitis, sepsis, hepatitis, hordeolum of eyelid, keratoconjunctivitis, pneumonia, acute bronchitis, acute bronchiolitis, cellulitis and abscess of mouth, local infection of the skin and subcutaneous tissue, arthritis and nephritis.

Varicella-related antiviral use was identified by extracting, for all eligible patients, all the prescriptions corresponding to ATC codes J05 (antivirals for systemic use), D06BB (antivirals for topical use), and S01AD (ophthalmological antivirals) with a hard-coded diagnosis of varicella.

Baseline (i.e. at the index-visit) patient characteristics such as gender, age group and presence of complications were used as categorical variables and presented as numbers (percentage) in the analysis. Mean, minimum and maximum age and proportion of males were presented for each of the categories. Proportion of patients with complications split per type of complications and age group was also calculated.

## Results

### Patient and disease characteristics and varicella-related complications

**Fig. 2:**
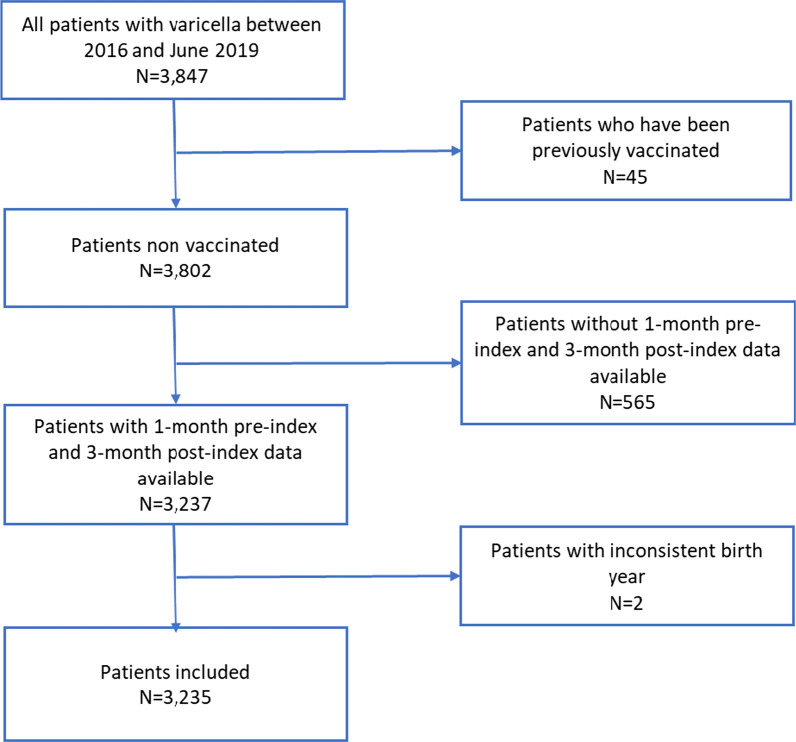
Selection flow chart

A total of 3847 patients with an index-visit for varicella were identified from the database in the indicated study period. Forty-five patients (1.1%) were discarded due to previous varicella vaccination and 565 due to insufficient pre-index and post-index data availability. Two more patients were excluded due to inconsistent birth year, resulting in a total of 3235 patients meeting the eligibility criteria for the study (Fig. 2).

Among the eligible patients, gender was distributed equally and the average age was 8.4 (SD: 13.7) years. 88.1% (n = 2849) were paediatric patients (defined as younger than 16 years old) and more than 75% of the patients were below 6 years old (Table 1). Only 8 patients had complications identified through the ICD-10-CM code for varicella (n = 6 with varicella meningitis and n = 2 with varicella encephalitis). When using the second approach for identification of varicella-related complications (as described in the “Materials and methods” section), 12.6% (n = 406) of the eligible patients had a concomitant diagnosis corresponding to a medical condition from the list derived from Blumental et al. [12] and Wolfson et al. [20] (Table 1). The most commonly identified complications over the 4-month period around the index-date were acute bronchitis (41.4%), skin and sub-tissue infections (39.4%), pneumonia (11.3%) and acute bronchiolitis (8.1%). Complications were more frequent in the 2–3 years age group (35.7% of the patients), followed by the 4–6 age group (20.0%), and the adult patients (17.2%) (defined as 16 years and older) (Table 2).

**Table 1:** Characteristics of patients with varicella at the index-visit

	N (%)	Male (%)
Total	3235 (100)	50.2
Presence of complications^a^		
Varicella with complications	406 (12.6)	51.0
Varicella without complications	2829 (87.5)	50.1
Age group		
0– year	452 (14)	49.6
2–3 years	1028 (31.8)	55.9
4–6 years	991 (30.6)	50.8
7–12 years	325 (10)	46.8
13–15 years	53 (1.6)	52.8
≥ 16 years	386 (11.9)	37.0

^a^Complications reported with/without hard-code link with varicella over the 1-month before and 3-month period after the index-visit. The list of complications was derived from Blumental et al. [12] and Wolfson et al. [20]

**Table 2:** Distribution of varicella-related complications per type and age group

Complications (ICD9 code)	Total n (%)	0–1 year n (%)	2–3 years n (%)	4–6 years n (%)	7–12 years n (%)	13–15 years n (%)	+ 16 years n (%)
Sepsis (A41)	2 (0.5)	0 (0)	0 (0)	0 (0)	0 (0)	0 (0)	2 (100)
Varicella meningitis (B01.0)	6 (1.5)	1 (16.7)	1 (16.7)	2 (33.3)	1 (16.7)	0 (0)	1 (16.7)
Varicella encephalitis, myelitis and encephalomyelitis (B01.1)	2 (0.5)	0 (0)	0 (0)	0 (0)	1 (50)	0 (0)	1 (50)
Hepatitis (B15–B19)	7 (1.7)	0 (0)	0 (0)	0 (0)	0 (0)	0 (0)	7 (100)
Hordeolum of eyelid (H00.0)	5 (1.2)	1 (20)	3 (60)	0 (0)	0 (0)	0 (0)	1 (20)
Keratoconjunctivitis (H19.1)	1 (0.2)	0 (0)	0 (0)	0 (0)	1 (100)	0 (0)	0 (0)
Pneumonia (J13–J18)	46 (11.3)	7 (15.2)	7 (15.2)	5 (10.9)	2 (4.3)	0 (0)	25 (54.3)
Acute bronchitis (J20)	168 (41.4)	30 (17.9)	66 (39.3)	40 (23.8)	12 (7.1)	1 (0.6)	19 (11.3)
Acute bronchiolitis, unspecified (J21.9)	33 (8.1)	18 (54.5)	9 (27.3)	1 (3)	1 (3)	1 (3)	3 (9.1)
Cellulitis and abscess of mouth (K12.2)	1 (0.2)	0 (0)	0 (0)	0 (0)	0 (0)	0 (0)	1 (100)
Local infection of the skin and subcutaneous tissue (L01–L08)	160 (39.4)	26 (16.3)	68 (42.5)	36 (22.5)	15 (9.4)	0 (0)	15 (9.4)
Arthritis (M00-M01)	1 (0.2)	0 (0)	0 (0)	0 (0)	0 (0)	0 (0)	1 (100)
Acute tubulo-interstitial nephritis (N10)	6 (1.5)	0 (0)	0 (0)	1 (16.7)	0 (0)	0 (0)	5 (83.3)
Total	406 (100)	75 (18.5)	145 (35.7)	81 (20.0)	33 (8.1)	2 (0.5)	70 (17.2)

### Antibiotic use

Over the 4-month follow up period, 27.3% (n = 833) of the patients with varicella were prescribed antibiotics, either systemic (19.8%) and/or topical (10.3%) (Table 3). For 11.6% (n = 374), antibiotics were prescribed at the index-visit itself (Additional file 1: Table S1) and for 5.3% (n = 172), antibiotics had been prescribed for varicella itself, with no hard-coded link to infectious complications. Among patients with complications, 63.5% (n = 258) were prescribed antibiotics. Conversely, these patients represented 31.0% (258 patients with complications who were prescribed antibiotics of the total of 833 patients with antibiotic prescriptions) of the total number of varicella patients being prescribed antibiotics (Table 3). The proportion of patients being prescribed antibiotics was the highest in patients with skin and subcutaneous infections (73.8%, with 60.6% receiving topical antibiotics) and in patients with acute bronchitis and pneumonia (36.9% and 32.6%, respectively, primarily systemic antibiotics) (Table 4). The antibiotics use consistently decreased with age, from 41.8% in patients aged 0–1 year, down to 13.7% in adult patients (Table 3). A similar trend was observed within the subgroup of patients with identified complications (Table 4). The three most commonly prescribed systemic antibiotics were amoxicillin alone (11.9% of varicella patients) or combined with enzyme inhibitor (beta-lactamase, 4.1%) and thiamphenicol (2.4%). The latter was administered via inhalation nonetheless, has been included with systemic antibiotics as per ATC-classification. Fusidic acid (7.1%) and tobramycin (1.4%) were the most prescribed topical antibiotics (Additional file 1: Table S2).

**Table 3:** Proportion of patients treated with antibiotics

	All indications (incl. varicella)			Varicella specific indication		
	On systemic AB (%)	On topical AB (%)	On any AB (%)	On systemic AB (%)	On topical AB (%)	On any AB (%)
All patients with varicella (N = 3235)	19.8	10.6	27.3	1.0	4.4	5.3
Presence of complications						
Varicella with complications (n = 406)	45.6	31.5	63.5	1.2	2.7	3.9
Varicella without complications (n = 2829)	16.1	7.6	22.1	1.0	4.7	5.5
Gender						
Male (n = 1625)	21.7	10.7	29.0	1.2	4.2	5.2
Female (n = 1610)	17.9	10.6	25.6	0.7	4.7	5.4
Age group						
0–1 year (n = 452)	32.5	17.5	41.8	1.8	6.0	7.7
2–3 years (n = 1028)	25.1	12.5	33.9	1.2	5.0	5.9
4–6 years (n = 991)	14.7	9.9	22.6	0.5	5.2	5.7
7–12 years (n = 325)	12.3	8.3	19.4	0.9	3.4	4.3
13–15 years (n = 53)	7.5	1.9	9.4	3.8	0.0	3.8
≥ 16 years (n = 386)	11.9	2.6	13.7	0.5	0.5	1.0

**Table 4:** Use of antibiotics (%) per type of complications and age group

	Total			0–1 year	2–3 years	4–6 years	7–12 years	13–15 years	+ 16 years
Complications (ICD9 code)	Topical	Systemic	Any	Any	Any	Any	Any	Any	Any
Sepsis (A41)	0.0	0.0	0.0	0.0	0.0	0.0	0.0	0.0	0.0
Varicella meningitis (B01.0)	0.0	0.0	0.0	0.0	0.0	0.0	0.0	0.0	0.0
Varicella encephalitis, myelitis and encephalomyelitis (B01.1)	0.0	0.0	0.0	0.0	0.0	0.0	0.0	0.0	0.0
Hepatitis (B15–B19)	0.0	0.0	0.0	0.0	0.0	0.0	0.0	0.0	0.0
Hordeolum of eyelid (H00.0)	60.0	0.0	60.0	0.0	100.0	0.0	0.0	0.0	0.0
Keratoconjunctivitis (H19.1)	0.0	0.0	0.0	0.0	0.0	0.0	0.0	0.0	0.0
Pneumonia (J13–J18)	0.0	32.6	32.6	71.4	85.7	60.0	50.0	0.0	0.0
Acute bronchitis (J20)	1.2	36.3	36.9	46.7	42.4	30.0	41.7	0.0	15.8
Acute bronchiolitis, unspecified (J21.9)	0.0	6.1	6.1	11.1	0.0	0.0	0.0	0.0	0.0
Cellulitis and abscess of mouth (K12.2)	0.0	0.0	0.0	0.0	0.0	0.0	0.0	0.0	0.0
Local infection of the skin and subcutaneous tissue (L01–L08)	60.6	19.4	73.8	88.5	70.6	83.3	73.3	0.0	40.0
Arthritis (M00–M01)	0.0	0.0	0.0	0.0	0.0	0.0	0.0	0.0	0.0
Acute tubulo-interstitial nephritis (N10)	0.0	16.7	16.7	0.0	0.0	100.0	0.0	0.0	0.0
Total	31.5	45.6	63.5	81.3	75.2	67.9	60.6	0.0	18.6

### Antiviral use

**Fig. 3:**
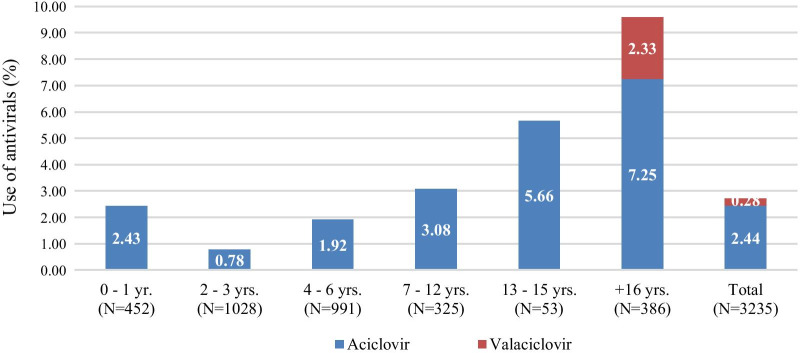
Use of antivirals for the treatment of varicella per age group

The use of antivirals specifically for varicella (i.e. with a hard-coded link to a varicella diagnosis) was documented in 2.7% (n = 88) of the study population (Fig. 3), with 94.3% being prescribed at the index-visit and the rest in the 3-month post-index (data not shown). The most commonly prescribed (systemic) antiviral drugs were aciclovir (2.44%; n = 79) and valaciclovir (0.28%; n = 9, only adult patients) (Fig. 3).

### Follow-up visits to GP

Finally, 4.7% of the patients (n = 152) had a follow-up visit to the GP with a diagnosis of varicella within the three-month post-index (Table 5). The mean number of all-cause GP visits (i.e. not necessarily related to varicella) in the 4-month study period was 1.8, with up to 14 GP follow-up visits for individual patients. GP follow-up visits were most important in patients younger than 1 year (2.2 follow-up visits), adults (2.4 follow-up visits) and patients with complications (2.6 follow-up visits) (Table 5). From these data, we calculated the annual incidence of GP consultation as follows: the incidence for a given year was the number of eligible patients with varicella, having their index-visit in that year, divided by the corresponding total number of patients in the database (regardless of the diagnosis). Thus, the total of 3235 patients with varicella included in this study imply a mean annual incidence of varicella-related GP consultation of 28.8 per 10,000 people.

**Table 5:** GP visits among varicella patients

	3-month post-index Varicella re-visit	Full study period All visits (incl. for varicella)	
	N (%) patients	Mean (SD) N visits	N visits min–max
All patients with varicella (N = 3235)	152 (4.7)	1.8 (1.2)	(1–14)
Presence of complications			
Varicella with complications (n = 406)	19 (4.7)	2.6 (1.4)	(1–10)
Varicella without complications (n = 2829)	133 (4.7)	1.7 (1.1)	(1–14)
Age group			
0 – 1 year (n = 452)	32 (7.1)	2.2 (1.5)	(1–10)
2 – 3 years (n = 1028)	59 (5.7)	1.9 (1.2)	(1–8)
4–6 years (n = 991)	33 (3.3)	1.6 (0.9)	(1–7)
7–12 years (n = 325)	11 (3.4)	1.4 (0.8)	(1–5)
13–15 years (n = 53)	4 (7.5)	1.6 (0.7)	(1–3)
≥ 16 years (n = 386)	13 (3.4)	2.4 (1.5)	(1–14)

## Discussion

The primary aim of this study was to describe the clinical burden of varicella management in terms of use of antibiotics, with or without complications, in a primary care setting in Belgium, where no UVV is currently implemented. The study was conducted using real-life data from the IQVIA Longitudinal Patient database, including GP data on approximately 460,000 Belgian residents. Secondary objectives aimed at describing other elements contributing to the burden of varicella in a primary care setting, in particular the use of antivirals and the number of visits to a GP.

12.6% of the > 3000 eligible patients experienced complications that may be related to varicella (according to Blumental et al. [12] and Wolfson et al. [20]). Also, over a quarter of all eligible patients were prescribed antibiotics during the 4-month follow-up period (19.8% systemic antibiotics and 10.6% topical antibiotics), with the highest proportion in younger age groups. Almost two-third of the patients experienced complications during the same follow-up period. The three most commonly prescribed antibiotics were amoxicillin, thiamphenicol and fusidic acid. Antiviral medication was prescribed in 2.7% of the patients. Of note, 1162 eligible patients had no prescriptions at all (data not shown), potentially indicating a mild disease course and potentially reflecting a limitation of the study that visits to pediatricians and hospitalizations are not captured in the data registry.

The percentage of systemic antibiotics use among varicella patients in this outpatient study (19.8%, Table 3) was higher than the 12.7% reported in a multi-country outpatient chart review study (it was 2.7% in Hungary and 12% in Poland, the two European countries involved) [20]. These differences are not merely a reflection of the national consumption rate of antimicrobials [24]. Nevertheless, the difference can be attributed to the fixed 4-month period for follow-up in our study, compared to the follow-up until the date of resolution of disease in the study of Wolfson et al. [20]. It is therefore possible that part of the reported antibiotic use in our study is not directly linked to varicella or varicella-related complications. Nevertheless, the complication rate in the chart review study was comparable to the rate found in our study (12.2% versus 12.6% in our study). Note, however, that patients included in the outpatient chart review study, were patients who visited an outpatient department or an emergency department for varicella, without hospitalisation. This population is likely not entirely comparable to the GP patient population included in our study. The actual impact of this difference on the use of antibiotics is difficult to assess. The relatively high complication rate in this primary care database study can be attributed to other comorbidities which we did not evaluate due to the retrospective nature of the study.

Several limitations of this study are important to mention. First, the study was conducted based on retrospective data obtained from a sample of 300 GPs, providing a coverage of approximately 2.5% of the full GP universe in Belgium. Although this database has been widely used in previous studies [21–23] and that it is nationally representative in terms of regions, gender and age, it can be debated whether the results are fully generalizable to the whole of Belgium. Another limitation resulting directly from the data source lies in the fact that it does not contain any data from paediatricians. It is possible that we are failing to capture a significant proportion of the paediatric population, given that the vast majority of varicella patients are children. Despite this limitation, almost 90% of our study population consisted of paediatric patients (defined as below 16 of age). In addition, due to the primary care setting, we have no data on the number of varicella-related hospitalisations. It is probable that a high number of severe complications (e.g. severe pulmonary infections, encephalitis,…) are not seen by the GP and thus not captured in this study. This might be an explanation for the relatively low numbers of varicella-related complications in the adult population (Table 2) and reflect a potential underestimation of severe complications in this study. The calculated mean annual incidence of GP consultations for varicella (28.8 per 10,000 people) is in line with the range of 28.8–35.7 cases per 10,000 people reported by Sabbe et al. [14] using a population from 2006 to 2010. Lastly, the data source of our study consists of anonymized electronic medical records, which were not originally designed for epidemiological studies. As with all real-world data sources, there may be inaccuracies or discrepancies in the details recorded in the database and given that all patients and physicians are kept anonymous to the researchers, it is not possible to make any ex-post validation.

The reported 12.6% varicella-related complications in our study is higher than described (2–6%) in some previous studies in primary care settings [12, 13]. This can possibly be explained by our alternative strategy for the identification of complications. The diagnosis codes used to identify relevant complications were based on the list of varicella-related complications, as reported in the studies of Blumental et al. [12] and Wolfson et al. [20]. The reason for moving to that strategy to retrieve complications was that only 8 patients with complications were identified through our initial approach (where complications were identified only through additional digits of the main varicella code), which was an obvious underestimation and a clear indication that these additional digits were not systematically used by the coder. On the other hand, our alternative approach may have resulted in an overestimation of the complications, by automatically assuming these concomitant diagnoses were a consequence of varicella. Given that the follow-up period to detect complications had been extended to a 4-month period around the index date (to be certain not to miss complications and also capture diagnoses that would have been confirmed with a delay in the patient file), it is likely that indeed some of the diagnoses identified as complications may not have been related to varicella and might be coincidental (e.g. high number of bronchitis in lower age group). It is however reasonable to assume that this is partly compensated by the fact that some of the more severe varicella-related complications may be seen in-hospital or by a paediatrician, and thus not appear in a GP database. Similarly, antibiotics and antivirals prescribed to patients in the outpatient and inpatient hospital setting are not included, possibly resulting in an underestimation.

The use of antibiotics in presence of varicella-related complications represented 29.2% of the total antibiotic use in our study (Additional file 1: Table S2). Nevertheless, it was not possible from the data to evaluate the (in)appropriateness of the antibiotic use as laboratory confirmations of bacterial infections were not captured in our database. To illustrate this, 36.9% of the eligible patients with a documented diagnosis of acute bronchitis were prescribed antibiotics (this proportion increased to 46.7% in infant patients, Table 4). This is not consistent with the guidelines of the Belgian Antibiotic Policy Coordination Committee (BAPCOC) that strongly advises against the prescription of antibiotics in the treatment of acute bronchitis in ambulatory care, neither in healthy paediatric patients nor in adult patients. Antibiotics are only indicated in bronchitis patients if concomitant bacterial pneumonia is suspected or in high risk adult patients [25]. These data suggest the use of antibiotics for indications that are not in line with the guidelines of BAPCOC, with potential important repercussions on the development of antimicrobial resistance.

In countries where UVV is already implemented, a significant decline in the varicella burden was observed [4]. Nevertheless, in our study, 1.1% of varicella cases had been previously vaccinated. Despite that varicella vaccination is not 100% effective, the vaccine program has been shown to result in a reduction of varicella-related cases, complications and hospitalisations in Greece, Spain, Italy and Germany (where it led to a 70% decline of varicella cases and 50% reduction in varicella-related hospitalisations) [4, 26–28]. In line with these observations, it can be expected that the number of visits to GPs and the use of antibiotics and antivirals would decrease concomitantly with the number of cases. Hence it is worthwhile to consider UVV in Belgium to reduce the burden of varicella, including development of complications and the use of antibiotics for management of varicella infections. Further cost-effectiveness studies are warranted to better understand long-term health and economic impact of UVV in Belgium.

## Conclusion

The burden of varicella in a primary care setting in Belgium is substantial, with high rates of complications as well as high rate of antibiotics use. To reduce the number of varicella cases and clinical and economic burden of varicella disease, including antibiotics use, policy makers may consider a UVV strategy [5, 29, 30].

## Supplementary Information

**Additional file 1**: **Table S1**. Proportion of varicella patients treated with antibiotics per period. **Table S2**. Treatment duration and prescribed number of distinct antibiotics by presence of complications over the full period.

## Data Availability

The data that support the findings of this study are available from IQVIA Consulting Solutions but restrictions apply to the availability of these data, which were used under license for the current study, and so are not publicly available. Data are however available from the authors upon reasonable request and with permission of IQVIA.
