# Supplementary material for: Assessing the use of antibiotics and the burden of varicella in Belgium using a retrospective GP database analysis
Source: BMC Infect Dis. 2021 Nov 11;21:1150. doi: 10.1186/s12879-021-06848-4 (PMC8582146; doi:10.1186/s12879-021-06848-4)
Supplement: Supplementary file 1 — Additional file 1: Table S1. Proportion of varicella patients treated with antibiotics per period. Table S2. Treatment duration and prescribed number of distinct antibiotics by presence of complications over the full period. [file 12879_2021_6848_MOESM1_ESM.doc]

Supplementary Table 1. Proportion of varicella patients treated with antibiotics per period

|  | **At index** | | | **Pre-index** | | | **Post-index** | | |
| --- | --- | --- | --- | --- | --- | --- | --- | --- | --- |
|  | **On systemic antibiotics (%)** | **On topical antibiotics (%)** | **On any antibiotics (%)** | **On systemic antibiotics (%)** | **On topical antibiotics (%)** | **On any antibiotics (%)** | **On systemic antibiotics (%)** | **On topical antibiotics (%)** | **On any antibiotics (%)** |
| *All patients with varicella (N=3,235)* | 4.3 | 7.8 | 11.6 | 5.5 | 0.8 | 6.2 | 13.1 | 2.4 | 14.6 |
| *Presence of complications* |  |  |  |  |  |  |  |  |  |
| Varicella with complications (n=406) | 11.3 | 21.2 | 30.3 | 13.3 | 2.5 | 15.3 | 31.3 | 9.6 | 36.5 |
| Varicella without complications (n=2,829) | 3.3 | 5.9 | 8.9 | 4.4 | 0.5 | 4.9 | 10.5 | 1.4 | 11.5 |
| *Gender* |  |  |  |  |  |  |  |  |  |
| Male (n=1,625) | 4.7 | 7.6 | 11.7 | 5.7 | 0.8 | 6.3 | 14.5 | 2.8 | 16.1 |
| Female (n=1,610) | 3.8 | 8.0 | 11.4 | 5.4 | 0.7 | 6.0 | 11.7 | 2.1 | 13.0 |
| *Age group* |  |  |  |  |  |  |  |  |  |
| 0 – 1 yr. (n=452) | 8.2 | 11.3 | 17.7 | 10.0 | 0.7 | 10.6 | 21.7 | 6.2 | 25.2 |
| 2 – 3 yrs. (n=1,028) | 4.8 | 9.1 | 13.7 | 7.5 | 1.1 | 8.5 | 16.7 | 2.9 | 18.6 |
| 4-6 yrs. (n=991) | 2.2 | 8.1 | 10.0 | 4.1 | 0.7 | 4.6 | 10.0 | 1.3 | 10.8 |
| 7-12 yrs. (n=325) | 4.3 | 6.5 | 10.2 | 2.8 | 0.3 | 3.1 | 7.1 | 1.8 | 8.6 |
| 13-15 yrs. (n=53) | 1.9 | 0.0 | 1.9 | 0.0 | 0.0 | 0.0 | 5.7 | 1.9 | 7.5 |
| >= 16 yrs. (n=386) | 3.9 | 1.6 | 5.2 | 1.8 | 0.8 | 2.3 | 7.3 | 0.3 | 7.3 |

Supplementary Table 2. Treatment duration and prescribed number of distinct antibiotics by presence of complications over the full period

| **Type** | **Molecule** | **ATCCODE** | **n (%) patients with AB (N=3,235)** | **Use in varicella related complications (%)** | **Use in non-varicella related complications (%)** | **Mean Duration (SD)** |
| --- | --- | --- | --- | --- | --- | --- |
| Systemic | Amoxicillin | J01CA04 | 386 (11.9) | 27.7 | 72.3 | 3.4 (5.2) |
| Systemic | Amoxicillin and Enzyme Inhibitor | J01CR02 | 132 (4.1) | 35.6 | 64.4 | 3.4 (4.1) |
| Systemic | Thiamphenicol, Combinations | J01BA52 | 79 (2.4) | 29.1 | 70.9 | 1.3 (1.4) |
| Systemic | Azithromycin | J01FA10 | 53 (1.6) | 34.0 | 66.0 | 3.9 (2.3) |
| Systemic | Clarithromycin | J01FA09 | 31 (1) | 48.4 | 51.6 | 4.6 (4.7) |
| Systemic | Flucloxacillin | J01CF05 | 26 (0.8) | 65.4 | 34.6 | 3.8 (6.1) |
| Systemic | Cefuroxime | J01DC02 | 9 (0.3) | 55.6 | 44.4 | 1.9 (0.3) |
| Systemic | Sulfamethoxazole and Trimethoprim | J01EE01 | 8 (0.2) | 12.5 | 87.5 | 1.3 (1) |
| Systemic | Fosfomycin | J01XX01 | 7 (0.2) | 28.6 | 71.4 | 5.9 (3.9) |
| Systemic | Thiamphenicol | J01BA02 | 6 (0.2) | 0.0 | 100.0 | 5.4 (1.3) |
| Systemic | Erythromycin | J01FA01 | 5 (0.2) | 20.0 | 80.0 | 5.5 (7.2) |
| Systemic | Clindamycin | J01FF01 | 5 (0.2) | 40.0 | 60.0 | 12 (4.7) |
| Systemic | Nitrofurantoin | J01XE01 | 4 (0.1) | 0.0 | 100.0 | 33.3 (19.2) |
| Systemic | Doxycycline | J01AA02 | 3 (0.1) | 0.0 | 100.0 | 10 (0) |
| Systemic | Cefadroxil | J01DB05 | 3 (0.1) | 33.3 | 66.7 | 1.8 (0.8) |
| Systemic | Ciprofloxacin | J01MA02 | 3 (0.1) | 33.3 | 66.7 | 35 (29.7) |
| Systemic | Lymecycline | J01AA04 | 2 (0.1) | 0.0 | 100.0 | 10 (0) |
| Systemic | Moxifloxacin | J01MA14 | 2 (0.1) | 50.0 | 50.0 | 10 (0) |
| Systemic | Minocycline | J01AA08 | 1 (0) | 0.0 | 100.0 | 21 (0) |
| Systemic | Ofloxacin | J01MA01 | 1 (0) | 0.0 | 100.0 | 5 (0) |
| Systemic | Levofloxacin | J01MA12 | 1 (0) | 0.0 | 100.0 | 14 (0) |
| **Systemic** | **Total** |  | **641 (19.8)** | **28.9** | **71.1** |  |
| **Systemic** | **Mean (SD) number of antibiotics prescribed** | | **1.2 (0.44)** | **1.3 (0.56)** | **1.15 (0.38)** |  |
| Topical | Fusidic Acid | D06AX01 | 230 (7.1) | 46.5 | 53.5 | 12.2 (7.6) |
| Topical | Tobramycin | S01AA12 | 46 (1.4) | 21.7 | 78.3 | 38.4 (35.5) |
| Topical | Combinations of Different Antibiotics | S01AA30 | 33 (1) | 12.1 | 87.9 | 22.1 (9.3) |
| Topical | Fusidic Acid | S01AA13 | 31 (1) | 32.3 | 67.7 | 60.8 (24.5) |
| Topical | Bacitracin | D06AX05 | 6 (0.2) | 0.0 | 100.0 | 16.7 (5.2) |
| Topical | Mupirocin | D06AX09 | 5 (0.2) | 60.0 | 40.0 | 6.3 (1.4) |
| Topical | Chlortetracycline | S01AA02 | 4 (0.1) | 25.0 | 75.0 | 41.7 (16.7) |
| **Topical** | **Total** |  | **344 (10.6)** | **37.2** | **62.8** |  |
| **Topical** | **Mean (SD) number of antibiotics prescribed** | | **1.02 (0.15)** | **1.03 (0.17)** | **1.02 (0.14)** |  |
| **Any** |  |  | **883 (27.3)** | **29.2** | **70.8** |  |
